# Malignant Bowel Obstruction in Advanced Gynecologic Cancers: An Updated Review from a Multidisciplinary Perspective

**DOI:** 10.1155/2018/1867238

**Published:** 2018-05-17

**Authors:** Yeh Chen Lee, Nazlin Jivraj, Catherine O'Brien, Tanya Chawla, Eran Shlomovitz, Sarah Buchanan, Jenny Lau, Jennifer Croke, Johane P. Allard, Preeti Dhar, Stephane Laframboise, Sarah E. Ferguson, Neesha Dhani, Marcus Butler, Pamela Ng, Terri Stuart-McEwan, Pamela Savage, Lisa Tinker, Amit M. Oza, Stephanie Lheureux

**Affiliations:** ^1^Division of Medical Oncology and Hematology, Princess Margaret Cancer Centre/University Health Network, Toronto, ON, Canada; ^2^Princess Margaret Cancer Centre, Toronto, ON, Canada; ^3^Division of General Surgery, University Health Network, Toronto, ON, Canada; ^4^Department of Surgery, University of Toronto, Toronto, ON, Canada; ^5^Joint Department of Medical Imaging, Princess Margaret Cancer Centre/University Health Network, Toronto, ON, Canada; ^6^Division of Interventional Radiology, University Health Network, Toronto, ON, Canada; ^7^Division of Supportive Care, Princess Margaret Cancer Centre/University Health Network, Toronto, ON, Canada; ^8^Radiation Medicine Program, Princess Margaret Cancer Centre/University Health Network, Toronto, ON, Canada; ^9^Division of Gastroenterology, University Health Network, University of Toronto, Toronto, ON, Canada; ^10^Division of Gynecologic Oncology, Princess Margaret Cancer Centre/University Health Network, Toronto, ON, Canada; ^11^Department of Obstetrics and Gynecology, University of Toronto, Toronto, Canada; ^12^Solid Tumour Oncology and Gattuso Rapid Diagnostic Centre, Princess Margaret Cancer Centre/University Health Network, Toronto, ON, Canada; ^13^Lawrence S. Bloomberg Faculty of Nursing, University of Toronto, Toronto, ON, Canada; ^14^Ambulatory Care-Solid Tumour Oncology, Princess Margaret Cancer Centre/University Health Network, Toronto, ON, Canada

## Abstract

Malignant bowel obstruction (MBO) is a major complication in women with advanced gynecologic cancers which imposes a significant burden on patients, caregivers, and healthcare systems. Symptoms of MBO are challenging to palliate and result in progressive decompensation of already vulnerable patients with limited therapeutic options and a short prognosis. However, there is a paucity of guidelines or innovative approaches to improve the care of women who develop MBO. MBO is a complex clinical situation that requires a multidisciplinary approach to ensure the appropriate treatment modality and interprofessional care to optimally manage these patients. This review summarizes the current literature on the different approaches targeting MBO management including surgical intervention, chemotherapy, total parenteral nutrition, and pharmacological treatment. In addition, the impact of MBO management on patients' quality of life (QOL) is examined. This article focuses on the challenges in developing evidence-based treatment guidelines for MBO and barriers in clinical trial design for MBO and proposes strategies to advance the MBO management. Collaboration is essential to design studies that may improve the overall care and quality of life for these patients. Prospective data are needed to inform clinical practice, establish a new benchmark for evidence-based MBO management, and better understand the biology of MBO.

## 1. Introduction

Malignant bowel obstruction (MBO) in women with advanced gynecologic cancer is common and a major clinical challenge as it is associated with protracted symptoms such as the inability to maintain oral intake, vomiting, and abdominal pain. Ovarian cancer is the dominant cause of MBO and the most lethal of all gynecologic malignancies [1–5]. In reported retrospective series, up to 51% of women [3–6] with recurrent ovarian cancer developed MBO and their median survival following MBO diagnosis ranged from 45 to 169 days [4, 5, 7–9]. Median survival was longer (124–408 days) [5, 7–10] for those selected patients who underwent palliative surgical intervention. Majority of these women would experience recurrent episodes of MBO over their short life expectancy [11].

Recognizing variations in clinical practice and the unmet need for evidence-based treatment, the International Conference on MBO and Clinical Protocol Committee established a unifying definition for MBO to advance research in this field [12]. The criteria to define MBO are (i) clinical evidence of bowel obstruction (history/physical/radiological examination), (ii) bowel obstruction beyond the ligament of Treitz, (iii) diagnosis of intra-abdominal cancer with incurable disease, or (iv) a diagnosis of non-intra-abdominal primary cancer with clear intraperitoneal disease [12].

Herein, we will examine recent advances in the literature targeting different modalities and aspects of MBO management, including palliative surgical intervention [13–28], chemotherapy [28–30], pharmacological management of symptoms [31–40], total parenteral nutrition (TPN) [30, 41–46], and quality of life (QOL) in patients with MBO.

## 2. Diagnosis of MBO

MBO can be partial or complete and can occur at single or multiple sites. Small bowel obstruction is more common than large bowel obstruction (61% versus 33%, resp.) [47, 48]. The majority of MBO occurs due to external compression or functional occlusion of the gastrointestinal tract from peritoneal carcinomatosis or tumor infiltration of bowel muscle/nerves [49]. In some cases, the etiology of bowel obstruction may be related to nonmalignant causes (albeit not the focus of this review) such as adhesions from previous surgery, intraperitoneal chemotherapy, radiation enteritis, or opioids [17, 48].

Patients with advanced gynecologic cancers may develop MBO at primary presentation or more commonly at the time of disease recurrence or progression. The timing of MBO presentation as well as the underlying disease histology and extent of cancer spread are important factors to consider in management decision. Presentation of MBO is often subacute with cardinal symptoms such as nausea, vomiting, pain, abdominal distention, and absence of stools or passage of gas [48]. These symptoms are a result of distention-secretion-motor activity of the compromised bowel perpetuating the process of MBO: (i) accumulation of gastric, pancreatic, and biliary secretions, (ii) reduced absorption of water and salt from the intestinal lumen, and (iii) increased secretion of water and salt into the lumen [48, 50]. The cumulative impact of these events is the appearance of intestinal edema, dilated bowel loops with gas and fluid retention, and increased endoluminal pressure [48]. These episodes may be intermittent in cases of partial MBO, and patients may describe passage of liquid stools due to bacterial liquefaction of the digestive content and intestinal hypersecretion [48, 49].

**Figure 1:**
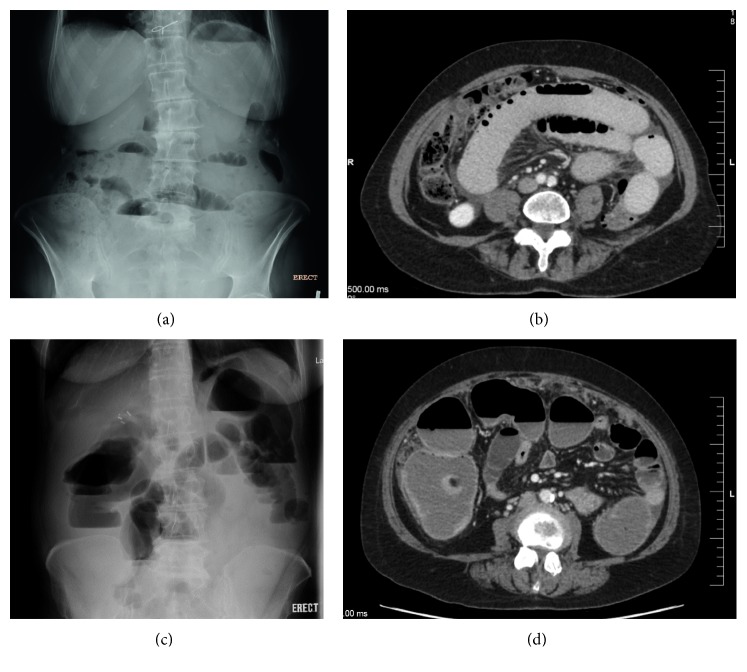
Radiographic images showing malignant bowel obstruction. (a) Abdominal radiograph in upright position showing multiple air-fluid levels consistent with small bowel obstruction (SBO). (b) Computed tomography (CT) confirms a high-grade SBO. (c) Abdominal radiographs in upright position showing large bowel obstruction (LBO). (d) CT demonstrates distended and fluid-filled large bowel loops concordant with LBO.

The diagnosis of MBO is established on clinical grounds and confirmed with abdominal imaging. Typical findings on abdominal radiographs seen in the upright position include distention of bowel loops with air-fluid levels in the segment proximal to the occlusion, as well as reduction in gas and stools in the segment distal to the occlusion (Figure 1) [51–53]. Plain abdominal radiographs have moderate sensitivity, ranging 40–80%, for detecting small bowel obstruction [51]. The absence of radiologic findings despite clinical symptoms suggestive of obstruction should not deter the clinician from the diagnosis as they may have functional bowel obstruction secondary to disseminated disease infiltration of the mesentery. Contrast computed tomography (CT) is more valuable as it provides diagnostic precision in identifying the site, etiology, and extent of obstruction and can confirm complications such as superimposed ischemia and intestinal perforation (Figure 1) [51]. Oral contrast agent administration in cases of suspected MBO is controversial, and the use of iodinated contrast medium (gastrografin) is preferred over barium as it is absorbable and provides similar radiological definition [52].

## 3. Surgical Intervention for MBO

Surgical intervention can be successful in reestablishing bowel function for selected MBO patients with good functional status and treatment options for the underlying cancer [10, 18, 48]. Large bowel obstruction is associated with significant morbidity and risk of perforation and death, and conservative management is not usually appropriate in this setting. The predominant surgical approach to large bowel obstruction consists of diverting stoma rather than primary resection and anastomosis or bypass [1, 54]. Small bowel obstruction without strangulation is mainly treated with conservative measures as it often relates to multifocal small bowel involvement secondary to peritoneal carcinomatosis [1, 54]. Only a minority would be considered for small bowel resection with anastomosis or internal bypass [1].

A systematic review of 868 patients with MBO showed that surgery was able to palliate obstructive symptoms (32–100%), enable resumption of a modified diet (45–75%), and facilitate successful patient discharge to home (34–87%) [54]. Similar results were found in another study examining treatment outcome for MBO under a multidisciplinary care model [10]. Compared to patients who received medical treatment, the surgical group had longer median survival (*p*=0.025), shorter hospitalization (*p*=0.02), more effective pain reduction (*p*=0.001), higher number of chemotherapy lines (*p*=0.02), and lower reobstruction rate (*p*=0.02) [10]. A prospective outcome analysis was performed on 26 patients with ovarian cancer who had undergone palliative procedures, including bowel diversion surgery (*n*=8), intestinal bypass/resection (*n*=6), and endoscopic procedures (*n*=12) [9]. The majority (*n*=23, 88%) reported overall symptomatic improvement or MBO resolution within 30 days, and ongoing symptom control at 60 days was achieved in 16 patients [9]. These aforementioned studies favor consideration of palliative surgery in highly selected patients with certain clinical characteristics, including good performance status, longer treatment-free interval, absence/small volume ascites, single-site disease, and albumin level [28, 55]. This is also in concordance with the recommendation by the European Association for Palliative Care that surgery should not be undertaken routinely in patients with poor performance status, intra-abdominal carcinomatosis, and massive ascites [47]. Only few reported series have investigated these clinical variables with mixed results, and it appears that collective assessment of the clinical variables into a risk-score system may be predictive of surgical outcome [4, 10, 56–58].

It is critical that discussions about realistic goals and limitations of surgery occur as it confers significant risk, with the operative mortality rate ranging from 6 to 32% and morbidity rate ranging from 7 to 44%, depending on type and setting (emergency versus elective) of the surgery [1, 13, 21, 22, 54]. There is also considerable risk of reobstruction (6–47%), hospital readmission (38–74%), and hospitalization for surgery which may consume a substantial portion of the patient's remaining life (11–61%) [54]. A Cochrane review examined the role of surgery in MBO secondary to advanced gynecologic and gastrointestinal cancer and included data from 43 studies with a total of 4265 participants [17]. No firm conclusion could be drawn due to the wide variability comparing different surgical procedures, the diverse definition of clinical outcome, heterogeneous clinical practice, and selection bias within these studies [17]. Therefore, the role for palliative surgery remains controversial and should only be considered in patients with more favourable disease factors and therapeutic options for their disease.

Less invasive approaches using self-expandable metallic stent (SEMS) for gastric outlet obstruction and left-sided colonic obstruction may be feasible in some cases of MBO. This procedure is less morbid compared to open surgery and is able to restore bowel function without the need of creating a stoma [22, 24]. The benefit of SEMS as a palliative procedure or as bridge to surgery has been well described with a lower overall morbidity and rate of temporary/permanent stoma [22–26, 59]. It was also recognized that the procedural success rate relied heavily on operator expertise and resources, and the overall complication rate can be as low as 3.4% for the risk of perforation and 0.5% for the risk of major bleeding [27].

For inoperable but symptomatic patients, venting gastrostomy may be placed to avoid the prolonged use of a nasogastric tube for digestive decompression, particularly in patients with protracted vomiting as their dominating symptom [20, 50, 60–62]. Placement of venting gastrostomy is shown to be feasible despite the inherently added risk of complications in patients with ascites [20, 60–62]. Prompt venting gastrostomy insertion can be advantageous in reducing the polypharmacy burden to control visceral symptoms, avoiding repeated hospital admissions for medical/nasogastric tube interventions, allowing consumption of modified diet for comfort, and facilitating sustained discharges to home or community palliative care unit [61].

## 4. Chemotherapy for MBO

The role of chemotherapy in MBO is to treat the underlying disease and requires careful consideration of the anticipated response and tolerability. There are very limited data in the literature given that patients with MBO are typically excluded from clinical trials. In addition, the majority of patients with MBO will have received multiple lines of chemotherapy and thus are unlikely to mount a clinically meaningful response resulting in the resolution of MBO [41, 46]. The type of chemotherapy prescribed for patients with advanced gynecologic cancers and MBO may include platinum-based therapy, taxane-based therapy, or gemcitabine [30]. Consideration of dose modification or a weekly regime is common as patients with MBO are at higher risk of toxicity and complications due to their general compromised nutritional state.

Two retrospective series investigated the provision of chemotherapy and TPN in patients with MBO and reported a median survival of 72 and 93 days, respectively [30, 41]. Patients who received chemotherapy and TPN following MBO diagnosis appears to have longer median survival compared to TPN alone [30, 41]. Chouhan et al. also reported resolution of small bowel obstruction in 10 of 82 patients, 5 of which were attributable solely to chemotherapy, and the remainder had received additional interventions such as surgery [30]. Of note, the study also included patients with advanced gastrointestinal cancer who were receiving first-line treatment for metastatic disease (albeit with chemosensitive disease). Overall, the currently available literature to support the use of chemotherapy in patients with advanced gynecologic cancers who developed MBO is still limited, and caution should be exercised when extrapolating data from larger MBO series that have a preponderance of nongynecologic cancers.

## 5. Total Parenteral Nutrition (TPN) in MBO

The use of TPN in patients with metastatic, incurable disease has been discouraged historically due to concerns regarding impact on QOL, increased risk of complications, and lack of proven benefit in the literature [30, 44, 45]. General guidelines have been established by expert committees, such as the European Society for Clinical Nutrition and Metabolism (ESPEN) and the American Society for Parenteral and Enteral Nutrition (ASPEN), although recommendations for patients with MBO remain vague [63, 64].

Studies examining the use of TPN in patients with advanced gynecologic cancer and MBO reported short median overall survival of 40–93 days [30, 41, 42, 65, 66]. In these studies, the rate of complications was highly variable, ranging from 4 to 54% [30, 41, 42], and they included predominantly catheter-related infections and less commonly deep venous thrombosis and TPN-related liver disease [67].

Embedded within these reported studies, there is invariably a subgroup of patients who survive for an extended period (24% survival at 6 months and 8% survival beyond 1 year), presumably as a result of TPN and relative disease stability based on biology [30, 41, 66–70]. It is reasonable to postulate that certain disease histology/biology (such as low-grade serous ovarian cancer) and the absence of cancer spread to visceral organs may correlate with better survival. There is however limited information to identify the characteristics that may predict such a sustained benefit from TPN. Bozzetti et al. suggested that the Glasgow prognostic score (GPS) of zero, Karnofsky performance status (KPS) >50, and tumor spread (local-locoregional disease) were significant prognostic factors of survival beyond 3 months following TPN [66]. Combining these three clinical variables may distinguish a patient subgroup whose survival at 6 months was 43.7% compared to 5%. A nomogram based on these parameters was developed enabling estimation of expected survival (3- and 6-month survivable probability) and needs further validation. In parallel with better understanding the biology of MBO and disease evolution, this proposed nomogram could help facilitate a balanced discussion and decision making for both health professionals and their patients. More work is underway to improve the proposed nomogram with inclusion of additional predictors and to establish its clinical utility [71].

The economic impact or cost effectiveness for the utility of TPN in patients with MBO is lacking and needs to be considered [70]. One systematic review demonstrated that the incremental cost-effectiveness ratio (ICER) for TPN in patients with inoperable MBO was high at £176,587 per quality adjusted life year (QALY; equivalent to CAD 312,071) [70]. In Canada, an intervention with an ICER greater than CAD 100,000 is typically viewed as a poor use of resources, although the threshold values are frequently debated and do not represent widely accepted standards [72]. In addition, these threshold values do not take into account the disease biology, treatment response, and reversibility of bowel obstruction.

Whilst it remains unclear how to best select the small subgroup of patients who derive extended benefit from TPN, balanced in-depth discussions and realistic expectations must be set with patients and family members early on to emphasize the limitations of TPN use in MBO and situations when TPN should be discontinued. Data on QOL are required to better inform decisions about the value of TPN in patients with MBO and what would be the minimal length of survival needed before benefit is likely to be experienced.

## 6. Pharmacological Management of MBO

Medical management in MBO is directed at reducing inflammation and endoluminal pressure and secretions as well as relief of pain and distressing symptoms. Polymodal medical treatment based on the combination of glucocorticoids, opioid analgesics, antiemetics, and antisecretory drugs can achieve good symptomatic control for MBO [10, 35, 39, 48, 73]. Most patients with MBO cannot tolerate oral medications; therefore, alternate methods of drug administration are considered such as intravenous, subcutaneous, and transdermal. Doses and choice of drugs are highly personalized and variable [10, 35, 69]. It is also necessary to adjust the medication regimen periodically depending on the trajectory of MBO and treatment response. There are palliative care guidelines established, such as the National Comprehensive Cancer Network [74], to aid clinicians to make appropriate prescriptions.

There are few studies investigating the use of steroids [31–35], somatostatin analogues [35–38], and olanzapine [40] in relieving the symptoms of MBO. A study investigating the use of dexamethasone (at a dose ranging up to 16 mg) in 35 patients showed a higher rate of spontaneous resolution of MBO in patients on dexamethasone compared to placebo, 37% versus 22%, respectively [31]. Another study assessed the use of methylprednisolone (240 mg or 40 mg versus placebo for 3 days) in 52 patients and showed trend for symptom improvement in the methylprednisolone group (59% versus 33.5%, *p*=0.08) [33]. Overall, there is a trend to support the use of steroids in MBO, and the side effects are generally well tolerated [34]. Concerns regarding prolonged use of glucocorticoids in this setting include infection risk, gastric ulceration, and mood swings and therefore should be rapidly tapered if minimal response is observed [73]. Recently, Obita et al. performed a systematic review on somatostatin analogues in the management of MBO and found no observed benefit of somatostatin analogues based on the highly variable primary outcome established by the seven eligible studies (a total of 427 patients) evaluated [36]. Nonetheless, somatostatin analogues appeared to be well tolerated with no dropouts due to toxicity as reported in the randomized controlled trials (RCTs) [36, 75].

Opioid analgesia is a common and effective medication used to palliate pain in advanced cancers, as supported by the WHO guidelines [76, 77]. Pain in MBO can be colicky (cramping and intermittent) or continuous in nature [12, 50]. There are very limited data on the optimal analgesic agent for MBO; however, experts favor the use of opioid analgesia given that it can be administered bypassing the oral route (intravenous, subcutaneous, sublingual, or transdermal) and the depressive effect on bowel motility may in fact relieve colicky pain [12, 50, 78].

## 7. Quality of Life (QOL)

Data on QOL and cost analysis were consistently lacking across the literature for MBO [26]. Such data are essential, particularly when the palliative management of patients with advanced disease is assessed. The resolution of MBO has typically been used as a surrogate marker for improved QOL. Bowel function recovery, and its measure for QOL, has been evaluated among patients undergoing stent or diverting colostomy [79, 80]. Whilst both methods were found to be effective in palliating symptoms of MBO, stent placement was associated with improved QOL related to gastrointestinal function [81].

A prospective study assessed the changes of QOL over three months in 35 patients with nongynecological cancers following the diagnosis of MBO, as measured by the Edmonton Symptom Assessment Scale (ESAS) and Rotterdam Symptom Checklist (RSCL) [82]. This showed a poor overall baseline QOL and subsequent significant improvements in QOL on many parameters with therapy (surgical/chemotherapy/supportive care) by one week and one month (*p* < 0.05), except for activity level and psychological functioning [82]. The overall improvement in QOL score plateaued after one month and remained similar at three months [82]. It is noteworthy that psychological distress continued to rank highly at three months despite the improvement in overall QOL.

One US-based study investigated QOL, nutritional status, and functional outcomes of 52 advanced cancer patients receiving TPN using validated methods such as EORTC-QLQ-C30, KPS, and Subjective Global Assessment (SGA) [83]. All of these patients also received active treatment including chemotherapy, radiation, or hormonal therapy [83]. This study demonstrated that TPN was associated with significant improvement in global QOL (increase of 6.3 points, *p* < 0.001), nutritional status (weight 1.3 kg, *p*=0.009), and functional status (KPS increase of 5.8, *p* < 0.001) each incremental month with the greatest benefit at three months [83].

## 8. Challenges in Optimizing Treatment for MBO

There is a paucity of RCT data for MBO due to inherent heterogeneity in the treatment paradigm in this already vulnerable patient population. Obtaining this level of evidence is challenging, and there are very limited clinical trials underway (Table 1). Most intervention studies reported were retrospective in nature targeting single modality of treatment within a specific MBO setting. Apart from confounding variables such as uncontrolled concurrent therapies, each intervention only targeted specific time points of a MBO episode and therefore did not inform the longitudinal MBO trajectory for these patients. One prospective study followed 35 patients with MBO for two years in a single institution and provided insight into the collaborative approach in multimodal interventions and symptom palliation over the multiple interjecting hospital admissions for these patients [69]. Yet, this study specifically excluded patients with gynecologic cancers [69]. Therefore, a prospective study examining the longitudinal MBO trajectory in advanced/recurrent gynecologic cancers is warranted.

Clinical trials designed for MBO have also proven to be difficult due to the complex nature of the clinical settings and variation in defining the primary outcome measure. There is an ongoing debate regarding what constitutes a clinically relevant study endpoint for symptom control in MBO. For instance, when should we expect to see the benefit of intervention, for how long should the benefit be sustained, and what magnitude of benefit would be considered clinically relevant for patients and caregivers [36]? The subacute and recurrent nature of MBO has also hindered the ability to appropriately assess the effectiveness of therapy. Therefore, the conduct of prospective clinical trials for MBO is necessary and requires a multidisciplinary team effort to define the complex care approach and improve treatment strategy.

## 9. Conclusions and Perspectives

MBO is a challenging complication of advanced gynecologic cancers, particularly in ovarian cancer. Clinical decision making involves complex considerations of different approaches with limited guidance available from the current literature. Only a small subset of patients with MBO appears to benefit from surgical interventions, and there is ongoing controversy with the use of chemotherapy and TPN, highlighting the need for further investigation. The majority of patients with MBO are treated with the medical management focusing on symptom control and quality of life. Early integration of palliative care is essential in the management of MBO symptoms and facilitates discussions addressing treatment expectations and goals of care [55, 73, 84].

Patients with MBO can be managed in an out-patient setting by the hospital specialist team with the support from community care services, such as home palliative care services, community nursing care, and domestic care services. This ambulatory model of care appears feasible and allows patients to be supported at home [85]. Patients with MBO can be closely monitored by a team of physicians and nurses who can manage their symptoms accordingly, thereby alleviating unnecessary emergency department visits. Education about MBO is also the key to empower patients (and their caregivers) to be confident in managing their symptoms and when to seek help.

**Figure 2:**
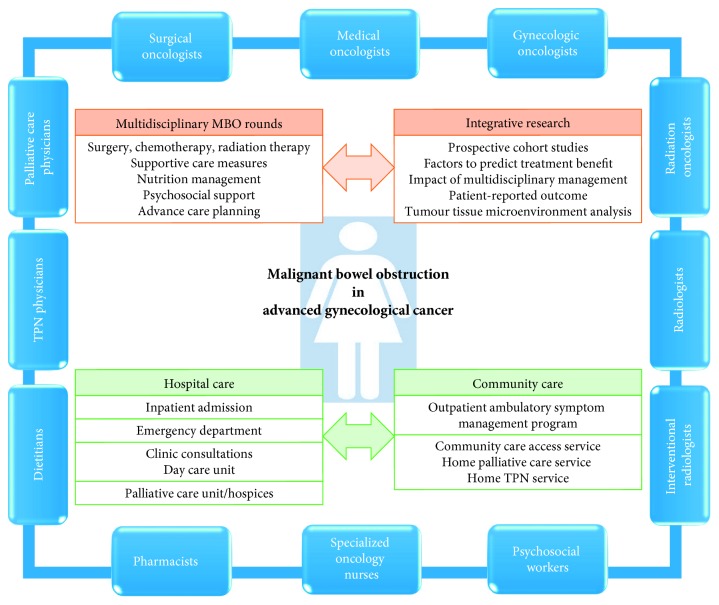
Interprofessional malignant bowel obstruction management team integrating clinical care and research.

In this complex care, establishing a multidisciplinary care platform to build consensus treatment strategies as patients navigate through different healthcare providers seems a logical approach to improve the care of this population, particularly with the goal of out-patient management. This promotes an effective communication across disciplines and ensures that patients receive consistent plans of care. In addition, the multidisciplinary platform will foster collaboration in designing and implementing best practice institutional processes and research proposals for MBO (Figure 2). A prospective study examining the MBO disease trajectory in patients with advanced gynecologic cancers under a dedicated multidisciplinary team approach is currently underway (NCT03260647, Table 1). Embedded within the study is the assessment of the ambulatory MBO management approach and quality of life using patient-reported outcomes. In the challenges in optimizing care for patients with MBO, a collaborative approach among the gynecologic cancer groups would be instrumental to establish best practice guidelines and implement feasible MBO research studies to optimize care for patients with MBO.

## Figures and Tables

**Table 1:** Current active clinical trials investigating malignant bowel obstruction.

Trials identifier	Trial name	Design	Intervention
NCT03260647	Risk-stratified multidisciplinary ambulatory management of malignant bowel obstruction in gynecological cancers (MAMBO)	Prospective observational study	Multidisciplinary MBO care program

NCT02365584	Quality of life in Patients with inoperable malignant bowel obstruction (QOL in IMBO)	Phase II, multicentre, RCT	Lanreotide with standard care versus standard care alone

NCT02275338	Study to assess efficacy and safety of lanreotide autogel 120 mg in treatment of clinical symptoms associated with inoperable malignant intestinal obstruction (IMIO)	Phase II, multicenter open label study	Lanreotide

NCT02270450	S1316, surgery or nonsurgical management in treating patients with intra-abdominal cancer and bowel obstruction	Phase III, RCT	Surgery versus nonsurgical management

NCT03150992	EDMONd–elemental diet in bowel obstruction	Phase II, open-label study	Dietary supplement: elemental 028 extra liquid
